# Cancer incidence and mortality trends in France over 1990–2018 for solid tumors: the sex gap is narrowing

**DOI:** 10.1186/s12885-021-08261-1

**Published:** 2021-06-24

**Authors:** G. Defossez, Z. Uhry, P. Delafosse, E. Dantony, T. d’Almeida, S. Plouvier, N. Bossard, A. M. Bouvier, F. Molinié, A. S. Woronoff, M. Colonna, P. Grosclaude, L. Remontet, A. Monnereau, the French Network of Cancer Registries (FRANCIM), Brice Amadeo, Isabelle Baldi, Simona Bara, Anne-Marie Bouvier, Véronique Bouvier, Marc Colonna, Gaëlle Coureau, Anne Cowppli-Bony, Sandrine Dabakuyo-Yonli, Tania d’Almeida, Laetitia Daubisse-Marliac, Gautier Defossez, Patricia Delafosse, Emmanuel Desandes, Pascale Grosclaude, Anne-Valérie Guizard, Brigitte Lacour, Bénédicte Lapôtre-Ledoux, Karima Hammas, Florence Molinié, Jean-Baptiste Nousbaum, Sandrine Plouvier, Camille Pouchieu, Michel Robaszkiewicz, Claire Schvartz, Brigitte Trétarre, Michel Velten, Anne-Sophie Woronoff

**Affiliations:** 1 Registre général des cancers de Poitou-Charentes, Pôle Biologie, Pharmacie et Santé Publique, CHU de Poitiers, Poitiers, France; 2 Université de Poitiers, Bâtiment D1, 6 rue de la Milétrie, 86073 Poitiers, France; 3 INSERM Centre d’Investigation Clinique CIC1402, Poitiers, France; 4 Réseau français des registres des cancers, Francim, Toulouse, France; 5 Direction des Maladies Non Transmissibles et des Traumatismes, Santé publique France, Saint-Maurice, France; 6 Service de Biostatistique-Bioinformatique, Pôle Santé Publique, Hospices Civils de Lyon, Lyon, France; 7 Université de Lyon, Lyon, France; 8 Université Lyon 1, Villeurbanne, France; 9 Équipe Biostatistique-Santé, Laboratoire de Biométrie et Biologie Évolutive, CNRS, UMR 5558, Villeurbanne, France; 10 Registre des cancers de l’Isère, CHU de Grenoble Alpes, Grenoble, France; 11 Registre des cancers de la Haute-Vienne, CHU de Limoges, Limoges, France; 12 Registre général des cancers de Lille et de sa région, Centre de Référence Régional en Cancérologie (C2RC), Lille, France; 13 Registre Bourguignon des cancers digestifs, Université de Dijon, Dijon, France; 14 Registre des tumeurs de Loire-Atlantique et Vendée, CHU de Nantes, Nantes, France; 15 Registre des tumeurs du Doubs, CHU de Besançon, Besançon, France; 16 EA 3181, Université de Bourgogne Franche-Comté, Besançon, France; 17 Registre des cancers du Tarn, Institut Claudius Regaud, Toulouse, France; 18 Institut Universitaire du Cancer de Toulouse Oncopole (IUCTO), Toulouse, France; 19 Registre des hémopathies malignes de Gironde, Institut Bergonié, Bordeaux, France; 20 INSERM U219 (équipe EPICENE), Institut de Santé Publique, d’Epidémiologie et de Développement (ISPED), Université de Bordeaux, Bordeaux, France

**Keywords:** Cancer, Incidence, Mortality, Registries, Sex, Trends

## Abstract

**Objective:**

To analyze trends in cancer incidence and mortality (France, 1990–2018), with a focus on men-women disparities.

**Methods:**

Incidence data stemmed from cancer registries (FRANCIM) and mortality data from national statistics (CépiDc). Incidence and mortality rates were modelled using bidimensional penalized splines of age and year (at diagnosis and at death, respectively). Trends in age-standardized rates were summarized by the average annual percent changes (AAPC) for all-cancers combined, 19 solid tumors, and 8 subsites. Sex gaps were indicated using male-to-female rate ratios (relative difference) and male-to-female rate differences (absolute difference) in 1990 and 2018, for incidence and mortality, respectively.

**Results:**

For all-cancers, the sex gap narrowed over 1990–2018 in incidence (1.6 to 1.2) and mortality (2.3 to 1.7). The largest decreases of the male-to-female incidence rate ratio were for cancers of the lung (9.5 to 2.2), lip - oral cavity - pharynx (10.9 to 3.1), esophagus (12.6 to 4.5) and larynx (17.1 to 7.1). Mixed trends emerged in lung and oesophageal cancers, probably explained by differing risk factors for the two main histological subtypes. Sex incidence gaps narrowed due to increasing trends in men and women for skin melanoma (0.7 to 1, due to initially higher rates in women), cancers of the liver (7.4 to 4.4) and pancreas (2.0 to 1.4). Sex incidence gaps narrowed for colon-rectum (1.7 to 1.4), urinary bladder (6.9 to 6.1) and stomach (2.7 to 2.4) driven by decreasing trends among men. Other cancers showed similar increasing incidence trends in both sexes leading to stable sex gaps: thyroid gland (0.3 to 0.3), kidney (2.2 to 2.4) and central nervous system (1.4 to 1.5).

**Conclusion:**

In France in 2018, while men still had higher risks of developing or dying from most cancers, the sex gap was narrowing. Efforts should focus on avoiding risk factors (e.g., smoking) and developing etiological studies to understand currently unexplained increasing trends.

**Supplementary Information:**

The online version contains supplementary material available at 10.1186/s12885-021-08261-1.

## Introduction

Cancer is a major public health issue worldwide and the first cause of death in France [1]. The monitoring of trends in incidence and mortality is a key resource for planning and assessing the impact of cancer control programs [2–4]. In France, national trends in cancer incidence and mortality are updated every 5 years and contribute to accurate knowledge of the burden of cancer and its changes over time [5–8]. These trends help public healthcare policy-makers to assess short- to medium-term prevention and care strategies. Descriptive analyses of such trends provide important information about the potential contribution of environmental exposures, primary preventive interventions, new treatments, and changing diagnostic and screening practices [9]. This exercise involves cautious interpretation of changing cancer incidence trends in concert with those in mortality [9]. While previous studies have challenged trends in France to identify environmental and system effects [5–7], no study has explicitly set out to focus on sex ratios of cancer incidence and mortality. Because of the different timing of exposure, sex gap is an epidemiological signature that we must consider, taking into account changing lifestyles and environmental exposures, which may lead to formulation of new hypotheses about the underlying risk factors and etiopathogenesis [10–12]. In France in 2015, 41% of all cancers were attributable to preventable risk factors with four leading contributors - tobacco, alcohol drinking, dietary factors and overweight or obesity [13–18]. While males have a historically higher prevalence of exposure to these risk factors than females, substantially variations have occurred over the last decades: more women tended to smoke [19, 20], historically high levels of alcohol consumption declined markedly [16, 20, 21] and prevalence of obesity has increased rapidly in men and women since 1990 [22, 23]. Changing population-level exposure to these modifiable risk factors may play a key role in changing cancer incidence. Understanding these changes by sex therefore seems interesting, the objective being to have the largest impact on reducing cancer incidence while prioritising risk-reduction policies.

The objective of this study is to provide an overview of recent patterns and long-term trends of cancer incidence and mortality in metropolitan France between 1990 and 2018, and to outline the main changes in terms of sex disparities. It considers 19 solid tumors (including sex-specific cancers), 8 subtypes, and the “all-cancers” entity (all solid tumors and hematological malignancies) as reporting an overall status of cancer burden.

## Material and methods

### Incidence, mortality and population data

Incidence data (1975–2015) were provided by the French population-based cancer registries (Francim network). Depending on the cancer site, the network is currently covering 19 to 22 French districts (*Département*); that is, 21 to 24% of the metropolitan population. The oldest registry started collecting data in 1975 and the most recent in 2009 (Supplementary Table S1). All malignant tumors (except non-melanoma skin cancer) are included and grouped according to the International Classification of Diseases for Oncology, 3rd Edition (ICD-O3) (Supplementary Table S2); the term “All cancers” refers to all malignant tumors, including hematological malignancies.

Mortality data (1975–2015) were provided by the *Centre d’épidémiologie sur les causes médicales de Décès* (CépiDc-Inserm). In this database, the causes of deaths are coded according to the International Classification of Diseases (8th to 10th revision, depending on the period). Data are available for all French metropolitan districts.

The numbers of person-years by annual age, year (year of diagnosis for incidence and year of death for mortality), and district were calculated from population census data (1975 to 2018) provided by the *Institut national de la Statistique et des Études Économiques* (Insee).

Data were analyzed from 1985 for incidence (to stabilize estimation in 1990) and from 1975 for mortality (to estimate long-term cohort indicator not presented in the present paper).

### Statistical modelling and indicators

The methodology used to obtain national incidence from local incidence data was detailed and validated in a dedicated paper [24]. Briefly, national incidence was estimated using incidence data alone (without correction with mortality as in older French studies on solid tumors) [5–7]. Incidence estimates were derived from a Poisson model where incidence rates were modelled by a bidimensional penalized spline of age and year of diagnosis plus a district random-effect. The national mortality rate was modelled by a bidimensional penalized spline of age and year of death. For incidence and mortality, the bidimensional model was compared with a simpler model (a model without age-year interaction and another model without year effect), using the Akaike Information Criterion [25]. Bidimensional penalized splines are innovative flexible models that allow the trends to vary smoothly with age; they are suitable to model simple or complex trends through penalization, which provides the “best” trade-off between fit and smoothness [25, 26].

Age-standardized incidence rates (ASIR) and mortality rates (ASMR) per 100,000 person-years were estimated using these models and the World Standard Population [27]. The trends were presented over 1990–2018 on the basis of projections for years 2016 to 2018. Projections were provided to ensure the most current estimates at the time of the publication, as well as projections referred to a short time period to improve their reliability. Trends in ASIR and ASMR were summarized by the average annual percent changes (AAPCs) over the period 1990–2018.

Sex gaps were indicated in 1990 and in 2018 using male-to-female rate ratios (relative) and male-to-female rate differences (absolute), for incidence and mortality, respectively. Male-to-female rate ratios were calculated with their 95% confidence interval by using the male age-standardized rates as the numerator and the female age-standardized rates as the denominator. A male-to-female rate ratio > 1 indicates that male incidence exceeds female incidence; whereas, a male-to-female rate ratio < 1 indicates that female incidence exceeds that of men and a male-to-female rate ratio = 1 indicates no sex difference (the same for mortality). Percent changes in rate ratios were calculated to report the main variations in the sex gap over 1990–2018 on a relative scale (the 1990 rate ratios served as the reference). A negative percent changes indicates that the sex gap narrowed and vice versa, except for cancers with female predominance (e.g. anus, skin melanoma, and thyroid gland) for which this is the opposite. In addition to relative sex differences, we calculated absolute differences by taking into account the difference between male and female age-standardized rates in 1990 and in 2018. Points of change in rate differences were calculated to quantify the sex gap over 1990–2018 on absolute scale (the 1990 rates served as the reference).

This novel methodology allowed accurate analyzes by age as well as incidence estimates by anatomical or histological subtype (see full results in Ref. [8]).

All analyzes were performed in R, release 3.4.3, using *gam* function from *mgcv* package [28].

### Cancer sites studied

Incidence and mortality were analyzed for all malignant cancers (all solid tumors and hematological malignancies) and for 19 malignant solid cancers (referring to 13 non-sex-specific cancers and 6 sex-specific cancers: two in men and four in women) (Supplementary Table S2).

Incidence trends are detailed by anatomical subsite for colorectal cancer (C18: colon, C19: rectosigmoid junction; C20: rectum, C21: anus), and by histological subtype for cancers of the esophagus and the lung (Supplementary Table S3). The latter were selected because of their epidemiological and clinical interest and because they provide a better understanding of their complex trends, which are related to specific risk factors, treatment modalities, or prognoses.

For prostate cancer, incidence indicators are provided for year 2015 and not 2018 because projection of the incidence of this cancer is highly uncertain.

Due to the high proportion of “uterus, not otherwise specified” in nearly 60% of death certificates, a specific statistical procedure was necessary to obtain the “observed” numbers of deaths for cervix and corpus uteri cancers [8, 29]. The proportions of cervix and corpus uteri respectively among all cancer uteri deaths were first estimated by age and year from registry data (by convolution of incidence and survival) and then applied to the observed number of cancer uteri death in France (corpus, uteri or unspecified). Once the numbers obtained, they were modelled like those of other sites, using bidimensional penalized splines.

## Results

### Estimated numbers of new cancer cases and deaths in metropolitan France in 2018

**Fig. 1:**
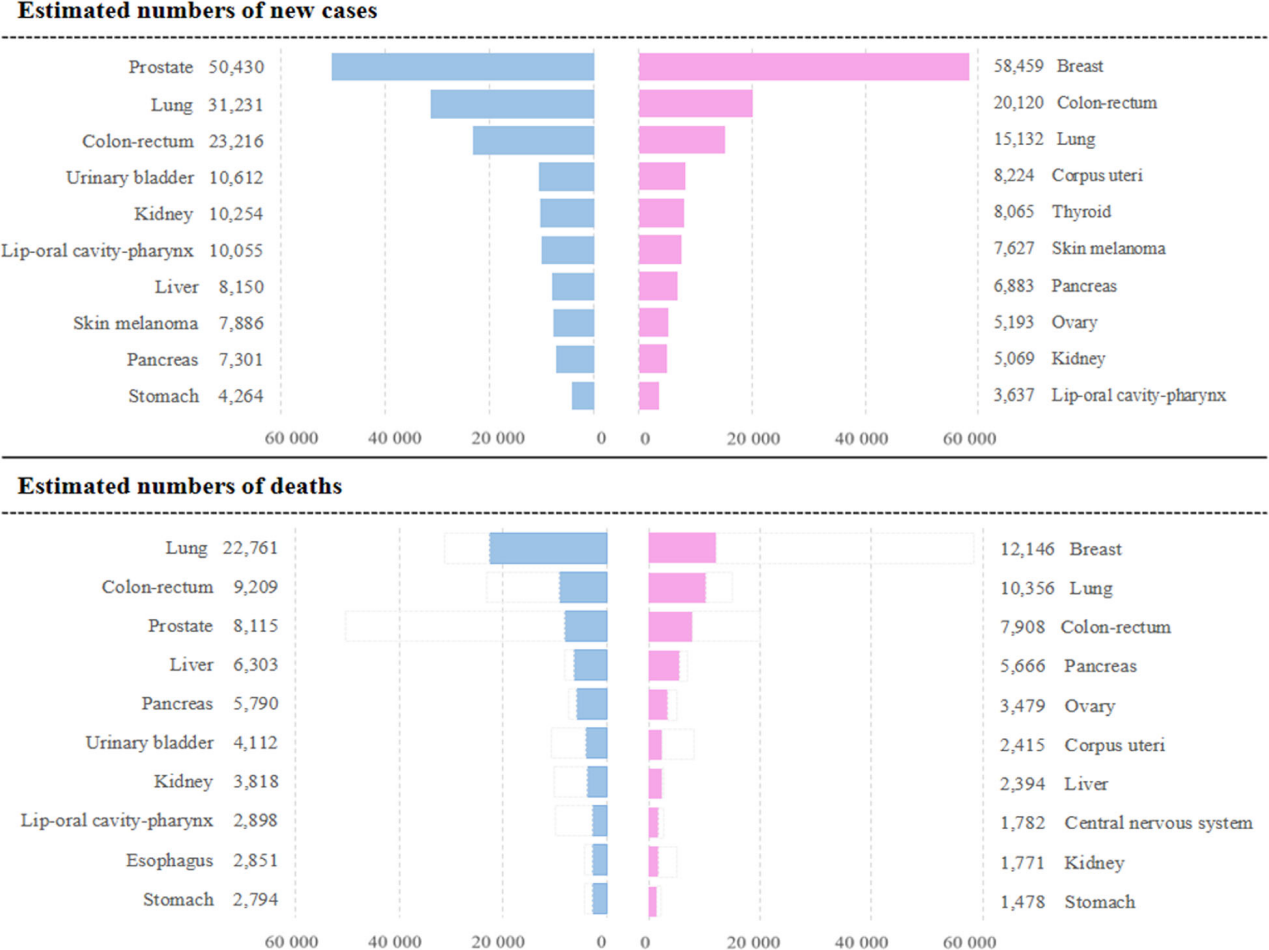
Ten leading cancer sites stemming from estimations of the numbers of new cases and deaths by sex, 2018, France. Note: The estimated number of new cases of prostate cancer relates to 2015 (last year of observation) and not 2018, due to the high level of uncertainty regarding the short-term incidence trends for this cancer

The estimation showed that 177,400 new cancer cases and 67,800 cancer deaths occurred in 2018 in France in women, versus 204,600 cases and 89,600 deaths in men. Figure 1 displays these estimates by sex for the ten most common cancers (See estimates for all sites in Supplementary Table S4). Breast cancer remained by far the most common cancer in women (33%), followed by colorectal cancer (11%) and lung cancer (9%). In men, the most common primary sites were the prostate (around 25%), the lung (15%) and colon-rectum (11%). Breast cancer was the leading cause of death from cancer in women (18%), followed by lung cancer (15%) and colorectal cancer (12%). Lung cancer remained the most common cause of death from cancer in men (25%), ahead of colorectal cancer (10%) and prostate cancer (9%).

### Trends in incidence and mortality between 1990 and 2018

Table 1 (for incidence) and Table 2 (for mortality) show, respectively, the ASIR and the ASMR (in 1990 and 2018), and the AAPCs (over 1990–2018), by sex, cancer site, as well as the male-to-female rate ratios and rate differences in 1990 and 2018 and their variations over 1990–2018. The 95% confidence intervals for ASIRs and ASMRs are reported in supplementary material (Tables S5 and S6).

**Table 1:** Age-standardized incidence rates and average annual percent change by sex, cancer site, and subtype with male-to-female (M/F) rate ratios and rate differences, France

	Men			Women			M/F					
	Age standardized rates^a^		Average annual percent change (AAPC) [95% CI^b^]	Age standardized rates^a^		Average annual percent change (AAPC) [95% CI^b^]	Relative differences			Absolute differences		
							Rate ratios [95% CI^b^]		Percent change in rate ratios	Rate differences		Points of change in rate differences
Cancer site or subtype	1990	2018	1990-2018	1990	2018	1990-2018	1990	2018	1990-2018	1990	2018	1990-2018
*All cancers (including hematological malignancies)*	320.7	330.2	0.1 [0.1; 0.2]	200.6	274.0	1.1 [1.1; 1.2]	1.6 [1.5;1.7]	1.2 [1.2;1.3]	-25%	120.1	56.2	-63.9
*Lip, oral cavity and pharynx*	38.6	18.3	-2.6 [-2.8; -2.5]	3.5	5.8	1.8 [1.5; 2.1]	10.9 [9.6;12.4]	3.1 [2.8;3.6]	-72%	35.1	12.5	-22.6
*Esophagus*	14.7	6.8	-2.7 [-3; -2.5]	1.2	1.5	0.9 [0.5; 1.3]	12.6 [10.6;15.1]	4.5 [3.8;5.4]	-64%	13.5	5.3	-8.2
Adenocarcinomas	1.2	2.8	2.9 [2.5; 3.4]	0.1	0.3	1.9 [0.9; 2.8]	8.3 [6.6;10.4]	11 [8.8;13.7]	33%	1.1	2.5	1.4
Squamous cell carcinomas	12.8	3.9	-4.1 [-4.4; -3.9]	0.9	1.2	0.9 [0.3; 1.4]	13.6 [11;16.7]	3.3 [2.6;4.1]	-76%	11.9	2.7	-9.2
*Stomach*	12.2	6.3	-2.3 [-2.5;-2.1]	4.6	2.7	-1.9 [-2.2; -1.6]	2.7 [2.4;3]	2.4 [2.1;2.7]	-11%	7.6	3.6	-4.0
*Colon-rectum*	40.0	34.0	-0.6 [-0.7; -0.5]	24.0	23.9	0 [-0.1; 0.1]	1.7 [1.6;1.7]	1.4 [1.3;1.5]	-18%	16.0	10.1	-5.9
Colon	22.6	20.7	-0.3 [-0.4; -0.2]	15.1	14.8	-0.1 [-0.2; 0.1]	1.5 [1.4;1.6]	1.4 [1.3;1.5]	-7%	7.5	5.9	-1.6
Rectum	17.0	12.7	-1.0 [-1.2; -0.9]	8.1	6.9	-0.5 [-0.7; -0.3]	2.1 [2;2.2]	1.8 [1.7;2]	-14%	8.9	5.8	-3.1
Anus	0.5	0.8	1.5 [0.7; 2.2]	0.9	2.4	3.4 [2.9; 3.9]	0.6 [0.5;0.7]	0.3 [0.3;0.4]	-50%	-0.4	-1.6	-1.2
*Liver*	8.0	12.5	1.6 [1.4; 1.8]	1.1	2.9	3.5 [3.1; 3.9]	7.4 [6.3;8.7]	4.4 [3.8;5.1]	-41%	6.9	9.6	2.7
*Pancreas*	5.2	11	2.7 [2.5; 2.9]	2.7	7.7	3.8 [3.6; 4.1]	2 [1.8;2.1]	1.4 [1.3;1.6]	-30%	2.5	3.3	0.8
*Larynx*	11.6	4.8	-3.1 [-3.4; -2.8]	0.7	0.7	0 [NC]^c^	17.1 [15.5;18.9]	7.1 [6.3;7.9]	-58%	10.9	4.1	-6.8
*Lung*	51.8	50.5	-0.1 [-0.2; 0]	5.4	23.2	5.3 [5.1; 5.5]	9.5 [8.6;10.5]	2.2 [2;2.4]	-77%	46.4	27.3	-19.1
Adenocarcinomas	8.9	26.2	3.9 [3.7; 4.1]	1.9	15.1	7.7 [7.4; 8.1]	4.7 [4.2;5.3]	1.7 [1.6;1.9]	-64%	7.0	11.1	4.1
Squamous cell carcinomas	25.9	11.3	-2.9 [-3.1; -2.7]	1.4	2.4	2.1 [1.6; 2.6]	19.1 [16.4;22.4]	4.7 [4;5.5]	-75%	24.5	8.9	-15.6
Small cell carcinomas	7.1	5.5	-0.9 [-1.2; -0.6]	0.8	2.7	4.4 [3.9; 5]	8.8 [7.5;10.5]	2 [1.7;2.4]	-77%	6.3	2.8	-3.5
*Skin melanoma*	4.8	14.2	4.0 [3.7; 4.2]	6.7	14.2	2.7 [2.5; 3]	0.7 [0.6;0.8]	1 [0.9;1.1]	43%	-1.9	0.0	1.9
*Breast*	-	-	-	72.8	99.9	1.1 [1; 1.2]	-	-	-	-	-	-
*Cervix uteris*	-	-	-	10.2	6.1	-1.8 [-2.1; -1.5]	-	-	-	-	-	-
*Corpus uteri*	-	-	-	10.6	11	0.1 [-0.1; 0.3]	-	-	-	-	-	-
*Ovary*	-	-	-	9.9	7.5	-1.0 [-1.2; -0.8]	-	-	-	-	-	-
*Prostate*	47.2	81.5^d^	2.2 ^d^ [2.1; 1.3]	-	-	-	-	-	-	-	-	-
*Testis*	4.3	8.7	2.6 [2.2; 2.9]	-	-	-	-	-	-	-	-	-
*Kidney*	10.6	17.1	1.7 [1.5; 1.9]	4.8	7.1	1.4 [1.2; 1.7]	2.2 [2;2.5]	2.4 [2.2;2.7]	9%	5.8	10.0	4.2
*Urinary bladder*	17.2	14.3	-0.7 [-0.8; -0.5]	2.5	2.4	-0.2 [-0.5; 0.1]	6.9 [6.2;7.6]	6.1 [5.4;6.8]	-12%	14.7	11.9	-2.8
*Central nervous system*	5.3	6.7	0.8 [0.6; 1.1]	3.7	4.5	0.6 [0.3; 1]	1.4 [1.3;1.5]	1.5 [1.4;1.6]	7%	1.6	2.2	0.6
*Thyroid gland*	1.7	5.6	4.4 [3.9; 4.8]	5.6	18.5	4.4 [4.1; 4.6]	0.3 [0.2;0.4]	0.3 [0.2;0.4]	0%	-3.9	-12.9	-9.0

^a^Incidence rates are expressed per 100,000 population and age-standardized to the 1960 standard world population (95% confidence intervals are reported in Supplementary Table S5)

^b^CI: confidence interval

^c^NC: 95% CI not calculable because the selected model does not include an effect for the year; p-value difference of AAPCs (men vs. women) not calculable either

^d^Incidence indicators for prostate cancer relate to 2015 (last year of observation) and not 2018 (see specific Materials and Methods), and thus the AAPC cover period 1990–2015 or 2010–2015, respectively

**Table 2:** Age-standardized mortality rates and average annual percent change by sex and by cancer site with male-to-female (M/F) rate ratios and rate differences, France

	Men			Women			M/F					
	Age standardized rates^a^		Average annual percent change (AAPC) [95% CI^b^]	Age standardized rates^a^		Average annual percent change (AAPC) [95% CI^b^]	Relative differences			Absolute differences		
							Rate ratios [95% CI^b^]		Percent change in rate ratios	Rate differences		Points of change in rate differences
Cancer site	1990	2018	1990-2018	1990	2018	1990-2018	1990	2018	1990-2018	1990	2018	1990-2018
*All cancers (including hematological malignancies)*	205.9	123.8	-1.8 [-1.8; -1.8]	90.1	72.2	-0.8 [-0.8; -0.8]	2.3 [2.3;2.3]	1.7 [1.7;1.7]	-26.1%	115.8	51.6	-64.2
*Lip, oral cavity and pharynx*	13.4	4.9	-3.5 [-3.7; -3.4]	1.3	1.2	-0.4 [-0.6; -0.2]	10.2 [9.9;10.5]	4.1 [3.9;4.4]	-59.8%	12.1	3.7	-8.4
*Esophagus*	11.3	4.3	-3.4 [-3.5; -3.3]	1.1	1.0	-0.3 [-0.5; -0.1]	10.6 [10.3;11]	4.3 [4.1;4.6]	-59.4%	10.2	3.3	-6.9
*Stomach*	9.0	3.9	-2.9 [-3; -2.8]	3.6	1.5	-3 [-3.2; -2.9]	2.5 [2.5;2.6]	2.6 [2.5;2.8]	4.0%	5.4	2.4	-3
*Colon-rectum*	18.2	11.5	-1.6 [-1.7; -1.6]	10.6	6.9	-1.6 [-1.6; -1.5]	1.7 [1.7;1.7]	1.7 [1.6;1.7]	0.0%	7.6	4.6	-3
*Liver*	10.4	9	-0.5 [-0.6; -0.4]	2.1	2.3	0.4 [0.3; 0.6]	5 [4.9;5.1]	3.9 [3.7;4]	-22.0%	8.3	6.7	-1.6
*Pancreas*	7.6	8.2	0.3 [0.2; 0.4]	3.9	5.5	1.2 [1.1; 1.3]	1.9 [1.9;2]	1.5 [1.4;1.5]	-21.1%	3.7	2.7	-1
*Larynx*	7.7	1.2	-6.3 [-6.5; -6.1]	0.3	0.2	-2.4 [-2.8; -1.9]	22.3 [21.2;23.5]	7 [6.1;8]	-68.6%	7.4	1	-6.4
*Lung*	48.2	34.7	-1.2 [-1.2; -1.1]	5.3	14.0	3.5 [3.4; 3.6]	9.1 [9;9.3]	2.5 [2.4;2.5]	-72.5%	42.9	20.7	-22.2
*Skin melanoma*	1.3	1.7	0.9 [0.7; 1.1]	1.0	1.0	0.2 [-0.1; 0.4]	1.3 [1.3;1.4]	1.6 [1.5;1.8]	23.1%	0.3	0.7	0.4
*Breast*	-	-	-	20.2	14.0	-1.3 [-1.4; -1.2]	-	-	-	-	-	-
*Cervix uteri*	-	-	-	3.1	1.7	-2.1 [-2.3; -1.9]	-	-	-	-	-	-
*Corpus uteri*	-	-	-	2.7	2.3	-0.5 [-0.6; -0.3]	-	-	-	-	-	-
*Ovary*	-	-	-	6.0	3.9	-1.5 [-1.7; -1.4]	-	-	-	-	-	-
*Prostate*	18.1	7.9	-2.9 [-3.0; -2.8]	-	-	-	-	-	-	-	-	-
*Testis*	0.4	0.2	-2.2 [-2.7; -1.6]	-	-	-	-	-	-	-	-	-
*Kidney*	4.6	5	0.3 [0.2; 0.4]	1.8	1.5	-0.6 [-0.8; -0.4]	2.5 [2.5;2.6]	3.2 [3.1;3.4]	28.0%	2.8	3.5	0.7
*Urinary bladder*	7.0	4.7	-1.4 [-1.5; -1.3]	1.3	0.9	-1.2 [-1.4; -1]	5.5 [5.3;5.6]	5.1 [4.8;5.4]	-1.8%	5.7	3.8	-1.9
*Central nervous system*	3.9	4.3	0.3 [0.2; 0.5]	2.5	2.7	0.3 [0.1; 0.5]	1.6 [1.5;1.6]	1.6 [1.5;1.7]	0.0%	1.4	1.6	0.2
*Thyroid gland*	0.4	0.2	-1.9 [-2.2; -1.5]	0.5	0.2	-3.4 [-3.7; -3]	0.7 [0.7;0.8]	1.1 [1;1.3]	57.1%	-0.1	0	0.1

^a^Mortality rates are expressed per 100,000 population and age-standardized to the 1960 standard world population (95% confidence intervals are reported in Supplementary Table S6); Deaths rates for cervix uteri and corpus uteri cancers were re-estimated (see specific Materials and Methods)

^b^CI: confidence interval

**Fig. 2:**
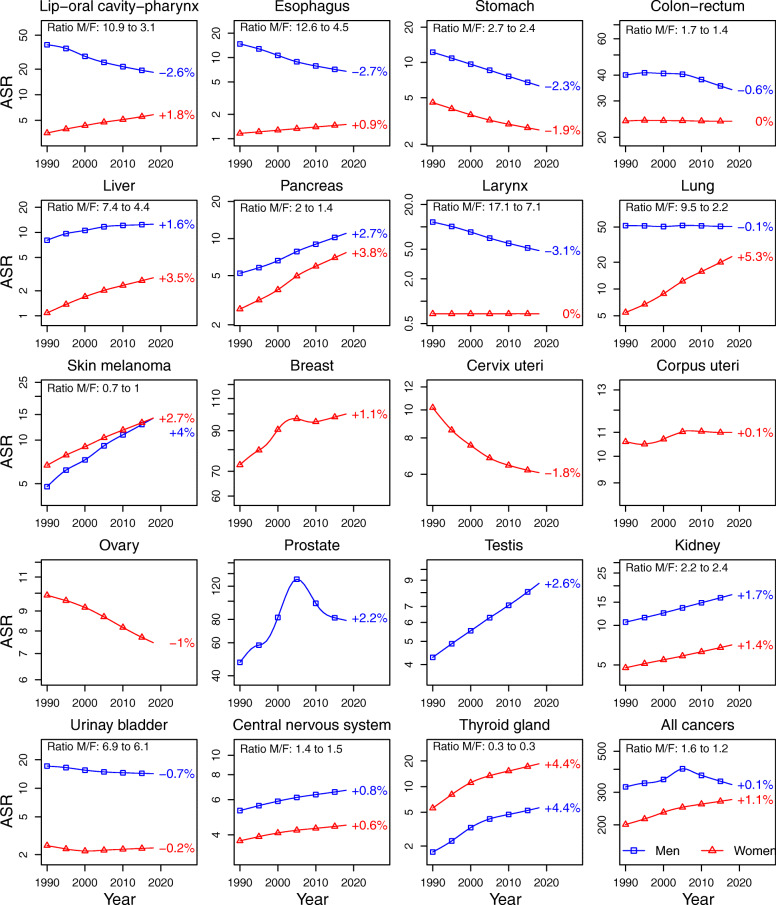
Trends in age-standardized incidence rates (log-scale) by sex for the main solid cancer sites and for all cancers, 1990–2018, France. Note: Cancer sites are displayed in the order in which they appear in the numerical list of ICD-O3 topography section (see details in Supplementary Table S2)

**Fig. 3:**
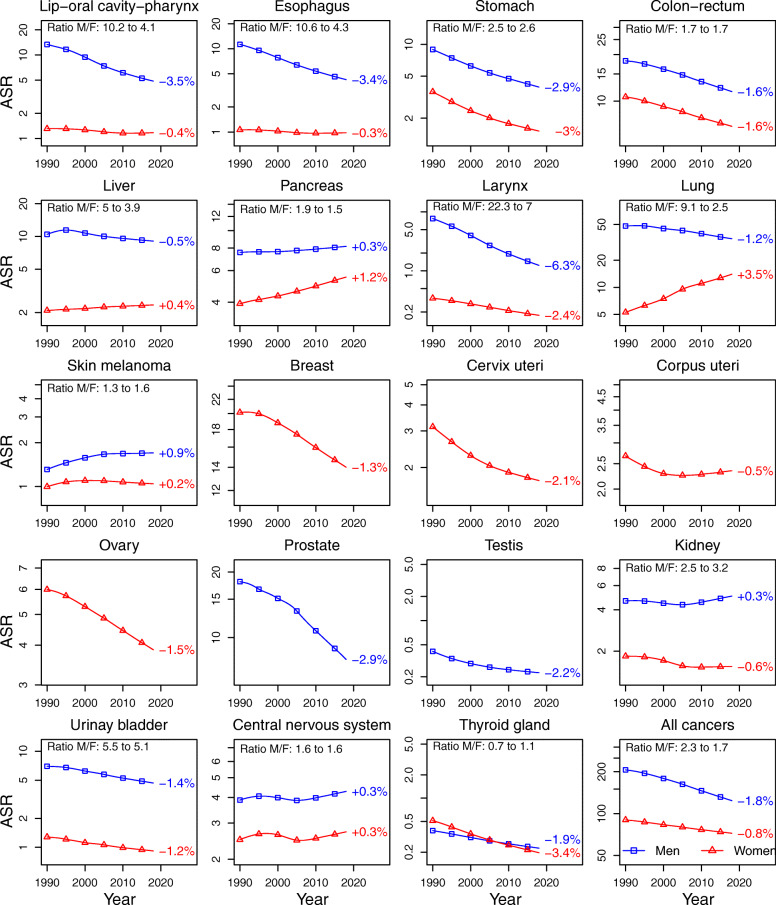
Trends in age-standardized mortality rates (log-scale) by sex for the main solid cancer sites and for all cancers, 1990–2018, France. Note: Cancer sites are displayed in the order in which they appear in the numerical list of ICD-O3 topography section (see details in Supplementary Table S2)

Synthetic view of trends in ASIR and ASMR by sex and cancer site are illustrated in Fig. 2 (for incidence) and Fig. 3 (for mortality).

#### All cancers

For all-cancers, the sex gap narrowed over 1990–2018 in incidence (1.6 to 1.2) and mortality (2.3 to 1.7). In men, all-cancer incidence rates were almost similar in 1990 and 2018, after having increased up until 2005 then decreased due to a sizable change in prostate cancer incidence. An estimation that excluded prostate cancers confirmed stable incidence in men over 1990–2018 (see Supplementary Figure S1).

#### Sex-specific cancer sites: prostate, testis, breast, corpus uteri, cervix uteri, ovary

Prostate, breast and testis cancer incidence increased over 1990–2018, while incidence rates remained stable for corpus uteri cancer and declined for ovary and cervix uteri cancers. Mortality declined for all six sex-specific cancers sites.

#### Cancer sites common to both sexes

##### Incidence.

Different patterns may be described on whether there is a decrease, stability or increase incidence in both sexes. Most of these changes result in a reduction in the sex incidence gap.

The largest decreases of the male-to-female rate ratios were for cancers of the lung (9.5 to 2.2), lip - oral cavity - pharynx (10.9 to 3.1), esophagus (12.6 to 4.5) and larynx (17.1 to 7.1). More than half of the male-to-female rate ratios decrease over 1990–2018 (percent change in rate ratios > 50%). In male, incidence remained stable (lung) or decreased (lip - oral cavity – pharynx, esophagus, larynx), while incidence increased in female, except for larynx cancer for which the incidence remained stable. In absolute scale, the largest reductions in sex incidence gaps were observed for cancers of the lip, oral cavity and pharynx, with male-to-female rate differences declining from 35.1 to. 12.5 per 100,000 person-years over 1990–2018, i.e. 22.6 points of change.

The sex gap narrowed more modestly for skin melanoma (0.7 to 1), cancers of the liver (7.4 to 4.4), pancreas (2 to 1.4) colon-rectum (1.7 to 1.4), urinary bladder (6.9 to 6.1) and stomach (2.7 to 2.4). For skin melanoma, the incidence was higher in women than in men in 1990 but increased more slowly in women, leading to similar incidence rates in 2018. For liver and pancreas cancers, incidence increased more rapidly in women than in men. However, the sex incidence gap remained stable or increased slightly in absolute scale (2.7 and 0.8 points, respectively). Favorable situations driven by decreasing incidence in men led to a narrowing sex gap for colon-rectum, urinary bladder and stomach cancers.

Sex incidence gaps were fairly stable for cancers of kidney (2.2 to 2.4), central nervous system (1.4 to 1.5) and thyroid gland (0.3 to 0.3) with increasing incidence trends in both sexes. The sex gap widened for thyroid gland cancer in absolute scale (9.0 points).

##### Mortality.

Sex mortality gaps narrowed for most of the cancers that showed a reduction in the sex incidence gap: cancers of the lung (9.1 to 2.5), larynx (22.3 to 7), lip, oral cavity and pharynx (10.2 to 4.1), esophagus (10.6 to 4.3), liver (5 to 3.9) and pancreas (1.9 to 1.5). The most important reduction in absolute scale was for lung cancer (22.2 points). Sex mortality gaps widened for kidney cancer (2.5 to 3.2) and skin melanoma (1.3 to 1.6) despite stabilizing or narrowing sex incidence gap, but with low variations in absolute scale (< 1 point). Sex mortality gaps remained stable for central nervous system (1.6 to 1.6), colon-rectum (1.7 to 1.7), urinary bladder (5.5 to 5.1) and stomach cancers (2.5 to 2.6).

#### Focus on anatomical and histological subsites

In men, the apparent overall stability of lung cancer incidence resulted from diverging trends of various histological subtypes (Table 1). The incidence of lung adenocarcinoma increased between 1990 and 2018 while that of small cell cancer and squamous cell carcinoma decreased. In women, the incidence rates of these three main histological subtypes increased, especially that of adenocarcinoma. The male-to-female incidence rate ratios fell from 4.7 in 1990 to 1.7 in 2018 for adenocarcinoma, 19.1 to 4.7 for squamous cell carcinoma, and 8.8 to 2.2 for small cell carcinomas. The sex incidence gap in absolute scale increased for adenocarcinoma (+ 4.1 points).

Similarly, there was a clear change in predominance regarding esophagus cancer between squamous cell carcinoma and adenocarcinoma in men, and an emerging predominance of adenocarcinoma in women, leading to an increase in the male-to-female incidence rate ratios for squamous cell carcinomas (8.3 to 11.0) an a decrease for adenocarcinoma (13.6 to 3.3).

When focusing on colon-rectum cancer subsites, the sex incidence gap remained stable for both colon and rectum cancers while it widened for anal cancer (0.6 to 0.3) due to more rapidly increased incidence in women than in men and already higher rates in 1990.

## Discussion

In France, sex gaps in cancer incidence and mortality have narrowed continuously over the last decades regarding all-cancers combined. In 2018, men were at higher risks of having or dying from cancer but as in other Western countries, these risks have been increasing steadily in women [30, 31]. Analysis of sex gaps by cancer sites and subtypes showed interesting underlying trends patterns.

### Women have closed the sex incidence gap for lung cancer, with mixed trends by histological subtypes

Changes in smoking behavior seem to be a major factor in the narrowing sex gap, given the substantial variations in incidence rates of tobacco-related cancers. For lung cancer, incidence increased dramatically in women, while it remained stable in men, in line with past exposures and changes of smoking habits. Women started smoking later than men (circa 1953) but the habit spread widely and rapidly until 1991, and then remained relatively stable [19, 20, 32, 33]. Tobacco smoking increased from 0.4 daily smoked cigarettes per women in 1953 to 3.7 in 1991, while men smoked approximately 8.6 cigarettes per day in 1953 until a peak in 1980 (9.6 cigarettes per day), then declined steadily (5.2 cigarettes per day in 2003). Impact of changes in smoking behavior is not yet visible for men in the recent trends in lung cancer, but a progressive decrease in incidence may be expected over the coming years (incidence recently started to decline in men under 50) [8]. However, the incidence in women will continue to increase among those born before the early 1990s, and further narrow the sex gap.

Trends in lung cancer by histological subtypes also provide interesting complementary information on sex disparities and the impact of smoking. Changes in cigarette design and composition (i.e. rise in filtered cigarettes and tobacco-specific N-nitrosamines) have probably driven smokers to deeper inhalation of small carcinogenic particles, leading to a shift from central tumors (squamous cell carcinoma) to peripheral tumors (adenocarcinoma) [34, 35], which is particularly obvious in men given their high initial rates. In contrast, the incidence of adenocarcinoma increased in a very worrying way in both sexes and the sex gap continued to widen regard the number of cases (i.e. in absolute scale). Adenocarcinoma remained the most frequent subtype among never-smokers; this suggests an involvement of competing risk factors (passive smoking, unhealthy diet, genetic susceptibility, environmental or occupational exposure to air pollutants --asbestos, fine particulate matter, radon gas, etc.) [36–38].

### Convergence of decreasing male and increasing female incidence rates for upper aerodigestive tract cancers

For upper aerodigestive tract cancers (i.e. lip - oral cavity - pharynx, esophagus and larynx), incidence showed contrary trends between men and women. On the one hand, alcohol consumption has been regularly decreasing since the fifties in France in all age categories and in both sexes. Adult per capita consumption in liters of pure alcohol has halved in 40 years, from 26 l in the early 1960s to 13 l in the early 2000s, with a downward trend that continued through 2018 [21, 39, 40]. The decreasing incidence of upper aerodigestive tract cancers in men may be partly explained by the synergistic effect of smoking and alcohol drinking reduction [21, 41]. On the other hand, in women, the rise in tobacco consumption undoubtedly played a key role in the rise of the incidence of these cancers, which may have concealed the roles of other risk factors [42, 43].

Concerning esophageal cancer, there was once again a clear shift from squamous cell carcinoma to adenocarcinoma in men, likely driven by the strong known association between tobacco and squamous cell carcinoma. Conversely, the increasing prevalence of obesity [22, 44] - with its close association with gastroesophageal reflux disease - might have played a major role in the increase of adenocarcinoma in both sexes [45].

### Increasing incidence trends in men and women: liver, pancreas, skin melanoma, kidney, thyroid gland, central nervous system

For pancreatic and liver cancers, the sex incidence gap narrowed due to more rapidly increasing incidence in women than in men, whereas the male-to-female differences in rates remained stable or increased. The rise in pancreatic cancer incidence contrasts sharply with the stable lung cancer rates in men, suggesting the contribution of other factors besides smoking. Likewise, historical trends of alcohol consumption seem to be an unlikely cause of increasing incidence trends in both sexes. The possible effect of an improvement in medical imaging on cancer incidence, especially in pancreatic cancers, cannot be ruled out but it should not have concerned women more than men. Apart from obesity-promoting diets, which may partly explained these trends in both sexes, as well as the spread of smoking among women, the causes remained largely unknown [46, 47].

Skin melanoma is an exception insofar as incidence was higher in women than in men in 1990, but more recently, its incidence has increased more rapidly in men, leading to similar incidence rate in 2018. Personal risk factors and exposure to ultraviolet radiation (from the sun and/or tanning beds) seem to be the most important factors. Nonetheless, public health policies regarding the screening of skin cancer since the late nineties may have played a role in increasing the rates together with longer lifetime exposure to ultraviolet radiation in men [48].

Regarding kidney, thyroid gland, and central nervous system cancers, the increasing incidence trends were rather similar in both sexes and led to almost stable sex gaps. Kidney cancer incidence remains higher in men, as is thyroid gland cancer in women. It is unlikely that associated exposures of tobacco [49] and obesity [50] are major factors in the male predominance for kidney cancer, as these exposures have been changing over time. In the same way, although female predominance in thyroid cancer is well established, there seems to be no real consensus as to why [51, 52]. The reasons of these sex disparities remain questionable. Nonetheless, improvements in medical practices and diagnostic imaging may have played a role in both, by increasing the number of early and fortuitous diagnoses of otherwise asymptomatic forms or small local tumors in both sexes (i.e. incidental diagnosis) [53]. In the case of kidney cancer, this explanation is debatable because of the slight increase in mortality in men over 2010–2018. For central nervous system cancers, etiological studies are still needed to clarify these rising trends in males and females alike by elucidating the potential role of environmental and occupational exposures (including pesticides and electromagnetic fields) [54].

### Stable or decreasing incidence trends in women vs. decreasing trends in men: Colon rectum, urinary bladder and stomach

Finally, the following encouraging situations have led to a narrowing sex gap. In the case of stomach cancer, both incidence and mortality rates decreased in male and female alike, mainly driven by the decreasing prevalence of *Helicobacter pylori* infections [42, 55]. For colon rectum and urinary bladder cancers, reductions in male-to-female rate ratio were caused solely by decreased male incidence, while female rates remained fairly stable. The French nationwide colorectal screening program has probably contributed to the decreasing trends in incidence and mortality through resection of precancerous lesions and detection of early-stage tumors [56]. However, the lack of decrease in colorectal cancer incidence in women is disturbing, even though analyses by subsites have reported recent slight declines in rectal cancer incidence. Note that the incidence of anal cancer, as a distinct disease entity compared to overall colorectal cancer, has shown increasing trends, with a female predominance that tends to increase over time. An explanation can be found in the increased exposure to high-risk oncogenic human papillomaviruses (HPV-HR) that accompanied changes in the sexual behaviors in women born after 1950 [51, 57]; This would also explain the slowed decrease in the incidence of cervical cancer in women [58]. Concerning urinary bladder cancer, the main known risk factors in Western countries are smoking and occupational exposures [59, 60]. Despite the changes in smoking habits in men and women, sex disparities remained very high across the study period, suggesting yet unidentified risk factors. Therefore, while variations in male-to-female rate ratios may clearly be correlated to changes in lifestyle factors having an impact on lung and upper aerodigestive tract cancers, the others patterns are somewhat less evident to interpret and merit further discussion and investigation.

### Strengths and limitations

The main strengths of this study were the use of high-quality population-based incidence data, and refinement of the trends by histological subtypes in the new method adopted for national estimates. Because histological codes are not found in mortality data and since the new approach does not use incidence/mortality ratio, this methodology could now be applied to estimate the incidence of various histological subtypes, which substantially refined epidemiological interpretations. Note that the approach to national cancer-incidence trends now use only local-registry data, which represent about 20% of the population. The key condition in our survey was that district cancer incidence should have the same mean and variability within the registry area and the whole country, and both assumptions were verified. In addition, trends could be estimated since 1990 despite incomplete histories in some registries. A detailed discussion of these aspects may be found in a dedicated paper [24].

Our study has several limitations. Addressing cancer burden by sex was interesting because male-to-female rate ratio are less likely to be affected by changes in diagnostic techniques, preventive strategies, tumor definitions or coding practices [61]. However, interpretation of male-to-female rate ratios remains subject to the effects of gender constructs, which influence the behavior of clinicians and patients by determining how, when, and why a person accesses medical care [62]. It may be of interest in future studies to provide further information on these determinants, which may affect the sexes disproportionately. Another limitation is that men have inherently higher risk of developing cancers than women, with a natural difference that might be ascribed to genetic and hormonal influences of biological sex [62]. Taking into account all potential risk factors remains an in-depth exercise. Our assessment of the explainibility of sex disparities remains focused on gender-related behaviors (e.g. smoking, lifestyle and nutritional habits) which might amplify this natural difference by producing epigenetic effects on biological sex. The majority of exposures underlying changes in male-to-female rate ratios are, at best, speculative for most cancers and remain to be elucidated.

Trends by histological subtypes were provided for only the main subtypes for lung and esophageal cancers, representing a potential limitation in trend interpretation. However, distributions of other histological types by period and by sex were carefully checked and remained close between 1990 and 2015 [8], which led us to suppose that any potential misclassification of other histological subtypes was limited and is unlikely to modify our conclusions.

Finally, future improvements should consider cancer stage at diagnosis and social deprivation, which are two important determinants of cancer incidence [63]. Cancer control plans require not only incidence and mortality data, but also survival data for better insights into the effectiveness of cancer services.

## Conclusion

In France, in 2018, men remained more at risk of developing or dying from most cancers, and they continue to bear a heavier burden related to tobacco and alcohol-related cancers. However, sex gap has been narrowing due to a significant rise in tobacco-related cancers in women and will probably continue to narrow, given the expected decline in lung cancer incidence in men, as well as the continuous decrease observed for upper aerodigestive tracts cancers. Nevertheless, preventable risk factors are unlikely to fully explain the increasing trends observed for common and/or lethal cancers in both sexes (liver, pancreas, kidney, central nervous system cancers, skin melanoma). That said, concerted efforts should be strengthened in order to reduce a series of modifiable risk factors usually related to lifestyle, and etiological research should be pursued in order to understand and determine underlying risk factors.

## Supplementary Information

**Additional file 1: Supplementary Table S1.** Incidence data used to estimate cancer incidence. **Supplementary Table S2.** Codes from the International Classification of Diseases used for the cancer sites reported. **Supplementary Table S3.** Codes from the International Classification of Diseases for oncology – Third Edition used for cancer subtypes (histological type). **Supplementary Table S4.** Estimated numbers of new cancer cases and deaths by sex with 95% CI, 2018, France. **Supplementary Table S5.** Estimated annual world age-standardized incidence rates with their confidence intervals by sex, cancer site, and subtype. **Supplementary Table S6.** Estimated annual world age-standardized mortality rates with their confidence intervals by sex and by cancer site. **Supplementary Figure S1.** Trends in age-standardized cancer incidence and mortality rates (log-scale) by sex, 1990–2018, France (all cancers excluding prostate and breast cancers).

## Data Availability

The data that support the findings of this study are available from the corresponding author upon reasonable request.

## Declarations

### Competing of interest

None declared.
