# Supplementary material for: Cancer incidence and mortality trends in France over 1990–2018 for solid tumors: the sex gap is narrowing
Source: BMC Cancer. 2021 Jun 24;21:726. doi: 10.1186/s12885-021-08261-1 (PMC8223369; doi:10.1186/s12885-021-08261-1)
Supplement: Supplementary file 1 — Additional file 1: Supplementary Table S1. Incidence data used to estimate cancer incidence. Supplementary Table S2. Codes from the International Classification of Diseases used for the cancer sites reported. Supplementary Table S3. Codes from the International Classification of Diseases for oncology – Third Edition used for cancer subtypes (histological type). Supplementary Table S4. Estimated numbers of new cancer cases and deaths by sex with 95% CI, 2018, France. Supplementary Table S5. Estimated annual world age-standardized incidence rates with their confidence intervals by sex, cancer site, and subtype. Supplementary Table S6. Estimated annual world age-standardized mortality rates with their confidence intervals by sex and by cancer site. Supplementary Figure S1. Trends in age-standardized cancer incidence and mortality rates (log-scale) by sex, 1990–2018, France (all cancers excluding prostate and breast cancers). [file 12885_2021_8261_MOESM1_ESM.docx]

**Supplementary material**

**Cancer incidence and mortality trends in France over 1990-2018 for solid tumors: sex gap is narrowing**

**Authors**

G. Defossez, Z. Uhry, P. Delafosse, E. Dantony, T. d’Almeida, S. Plouvier, N. Bossard, A.M. Bouvier, F. Molinié, A.S. Woronoff, M. Colonna, P. Grosclaude, L. Remontet, A. Monnereau and the French Network of Cancer Registries (FRANCIM)

**Supplementary Table S1.** Incidence data used to estimate cancer incidence.

**Supplementary Table S2.** Codes from the International Classification of Diseases used for the cancer sites reported.

**Supplementary Table S3.** Codes from the International Classification of Diseases for oncology – Third Edition used for cancer subtypes (histological type).

**Supplementary Table S4.** Estimated numbers of new cancer cases and deaths by sex with 95% CI, 2018, France.

**Supplementary Table S5**. Estimated annual world age-standardized incidence rates with their confidence intervals by sex, cancer site, and subtype.

**Supplementary Table S6**. Estimated annual world age-standardized mortality rates with their confidence intervals by sex and by cancer site.

**Supplementary Figure S1**. Trends in age-standardized cancer incidence and mortality rates (log-scale) by sex, 1990-2018, France (all cancers excluding prostate and breast cancers).

**Table S1**. Incidence data used to estimate cancer incidence.

| Département | Type of registry | Years with available data |
| --- | --- | --- |
| 08- Ardennes | Thyroid | 1975-2015 |
| 14- Calvados^a^ | General | 1978-2015 |
| 16- Charente | General | 2008-2015 |
| 17- Charente-Maritime | General | 2008-2015 |
| 21- Côte d’Or | Digestive tract | 1976-2015 |
|  | Hematopoietic^b^ | 1980-2015 |
|  | Breast | 1982-2015 |
|  | Cervix uteri, corpus uteri | 1982-2013 |
| 25- Doubs | General | 1978-2015 |
| 29- Finistère | Digestive tract | 2005-2015 |
| 33- Gironde | Central nervous system | 2000-2015 |
|  | Hematopoietic^b^ | 2002-2015 |
|  | General (other sites) | 2005; 2008-2015 |
| 34- Hérault | General | 1987-2015 |
| 38- Isère | General | 1979-2015 |
| 44- Loire-Atlantique^c^ | Breast, colon, rectum | 1991-2015 |
|  | General (other sites) | 1998-2015 |
| 50- Manche^a^ | General | 1994-2015 |
| 61- Orne^a^ | Hematopoietic^b^ | 2002-2015 |
| 51- Marne | Thyroid | 1975-2015 |
| 67- Bas-Rhin | General | 1975-2013 |
| 68- Haut-Rhin | General | 1988-2015 |
| 71- Saône-et-Loire | Digestive tract | 1982-2015 |
| 79- Deux-Sèvres | General | 2008-2015 |
| 80- Somme | General | 1982-2015 |
| 81- Tarn | General | 1982-2015 |
| 85- Vendée | General | 1998-2015 |
| 86- Vienne | General | 2008-2015 |
| 87- Haute-Vienne | General | 2009-2015 |
| 90-Territoire de Belfort | General | 2007-2015 |
| LIL- Lille area | General | 2008-2015 |

^a^ The registry of hematopoietic cancers of Basse-Normandie covers the following French Départements: Calvados, Manche and Orne for the 2002-2015 period.

^b^ Data from hematopoietic cancers registries only contributed to the estimations of all cancers.

^c^ The registry of Loire-Atlantique was specialized in breast and colorectal cancers until 1997; it became a general cancer registry in 1998.

**Table S2**. Codes from the International Classification of Diseases used for the cancer sites reported.

| **Cancer sites** | **Incidence** | | **Mortality** | | |
| --- | --- | --- | --- | --- | --- |
|  | Topography  (ICD-O-3) | Morphology^1^  (ICD-O-3) | 1975-1978  (CIM-8) | 1979-1999  (CIM-9) | 2000-15  (CIM-10) |
| Lip-oral cavity-pharynx | C00-C14 | All ^1^ | 140-149 | 140-149 | C00-C14 |
| Esophagus | C15 | All ^1^ | 150 | 150 | C15 |
| Stomach | C16 | All ^1^ | 151 | 151 | C16 |
| Colon-rectum | C18-21 | All ^1^ | 153,154 | 153,154 | C18-21 |
| Liver | C22 | All ^1^ | 155 | 155 | C22 |
| Pancreas | C25 | All ^1^ | 157 | 157 | C25 |
| Larynx | C32 | All ^1^ | 161 | 161 | C32 |
| Lung | C33-34 | All ^1^ | 162 | 162 | C33-34 |
| Skin melanoma | C44 | 8720-8780 | 172 | 172 | C43 |
| Breast ^2^ | C50 | All ^1^ | 174 | 174 | C50 |
| Cervix uteri | C53 | All ^1^ | 180-182^3^ | 179-182^3^ | C53-55^3^ |
| Corpus uteri | C54 | All ^1^ |  |  |  |
| Ovary | C56,  C570-574 | All ^1^ | 183 | 183 | C56, C570-574 |
| Prostate | C61 | All ^1^ | 185 | 185 | C61 |
| Testis | C62 | All ^1^ | 186 | 186 | C62 |
| Kidney | C64-C66, C68 | All ^1^ | 189 | 189 | C64-C66, C68 |
| Urinary bladder | C67 | All ^1^ | 188 | 188 | C67 |
| Central nervous system | C70-72 | All ^1^ | 191,192 | 191,192 | C70-72 |
| Thyroid gland | C73 | All ^1^ | 193 | 193 | C73 |
| All cancers | C00-C80 | All ^4^ | 140-208 | 140-208 | C00-C97 |

^1^ Hematological malignancies were excluded from solid tumors

^2^ Breast cancer has been studied in women only

^3^ Deaths from cervix uteri and corpus uteri cancers were re-estimated (see specific comments in Materials and Methods)

^4^ All cancers, including hematological malignancies, excluding non-melanoma skin cancers

**Table S3**. Codes from the International Classification of Diseases for oncology – Third Edition used for cancer subtypes (histological type).

| **Histological subtypes** | **Topography ^1^** | **Morphology** |
| --- | --- | --- |
| **Esophagus** | C15 |  |
| Squamous cell carcinomas |  | 8051, 8070-78, 8082-8084, 8123 |
| Adenocarcinomas |  | 8140-41, 8143-44, 8190, 8200-01, 8210-11, 8260-63, 8310, 8320, 8480-81, 8490, 8550, 8570, 8576 |
| Other |  | Other ^2^ |
| Tumors without cytological or histological confirmation |  | 8000 |
| **Lung** | C34 |  |
| Small cell carcinomas |  | 8002, 8041-45 |
| Squamous cell carcinomas |  | 8050-52, 8070-76, 8083-8084 |
| Adenocarcinomas |  | 8140-41, 8143-44, 8180, 8190, 8201, 8210-11, 8230, 8250-55, 8260, 8263, 8265, 8290, 8310, 8323, 8333, 8410, 8440-41, 8470, 8480-81, 8490, 8508, 8522, 8550-51, 8570, 8572, 8574, 8576 |
| Other |  | Other ^2^ |
| Tumors without cytological or histological confirmation |  | 8000 |

^1^ Restricting topographies from the main site

^2^ All other morphologies excluding hematological malignancies and out of 8000

Note: Subtypes in gray were not considered for estimations. All morphological subsites are new compared to the previous study.

**Table S4**. Estimated numbers of new cancer cases and deaths by sex, 2018, France.

|  | Estimated new cases | | | |  | Estimated deaths | | | |
| --- | --- | --- | --- | --- | --- | --- | --- | --- | --- |
| Cancer site | Men | 95% CI | Women | 95% CI |  | Men | 95% CI | Women | 95% CI |
| Lip, oral cavity and pharynx | 10055 | [9085;11130] | 3637 | [3350;3950] |  | 2898 | [2813;2989] | 924 | [876;972] |
| sophagus | 4251 | [3709;4873] | 1194 | [1067;1339] |  | 2851 | [2766;2940] | 874 | [831;922] |
| Stomach | 4264 | [3982;4567] | 2293 | [2096;2514] |  | 2794 | [2717;2871] | 1478 | [1427;1531] |
| Colon-rectum | 23216 | [22381;24083] | 20120 | [19463;20794] |  | 9209 | [9053;9369] | 7908 | [7769;8054] |
| Liver | 8150 | [7293;9110] | 2430 | [2197;2689] |  | 6303 | [6172;6443] | 2394 | [2316;2479] |
| Pancreas | 7301 | [6871;7752] | 6883 | [6405;7398] |  | 5790 | [5677;5907] | 5666 | [5548;5785] |
| Larynx | 2753 | [2516;3013] | 407 | [384;432] |  | 819 | [777;862] | 131 | [116;145] |
| Lung | 31231 | [29219;33381] | 15132 | [14069;16277] |  | 22761 | [22503;23021] | 10356 | [10164;10556] |
| Skin melanoma | 7886 | [7098;8765] | 7627 | [7021;8279] |  | 1135 | [1084;1191] | 840 | [798;883] |
| Breast | - |  | 58459 | [56552;60434] |  | - |  | 12146 | [11969;12323] |
| Cervix uteri | - |  | 2920 | [2667;3193] |  | - |  | 1117 | [1069;1166] |
| Corpus uteri | - |  | 8224 | [7678;8812] |  | - |  | 2415 | [2340;2495] |
| Ovary | - |  | 5193 | [4899;5504] |  | - |  | 3479 | [3385;3576] |
| Prostate | 50430^1^ | [48506;52434] | - |  |  | 8115 | [7972;8262] | - |  |
| Testis | 2769 | [2513;3057] | - |  |  | 86 | [78;101] | - |  |
| Kidney | 10254 | [9662;10883] | 5069 | [4717;5446] |  | 3818 | [3711;3924] | 1771 | [1703;1839] |
| Urinary bladder | 10626 | [9933;11366] | 2448 | [2245;2671] |  | 4112 | [4012;4221] | 1223 | [1172;1276] |
| Central nervous system | 3280 | [3106;3466] | 2606 | [2449;2776] |  | 2346 | [2265;2431] | 1782 | [1710;1855] |
| Thyroid gland | 2600 | [2230;3030] | 8065 | [6877;9458] |  | 159 | [146;175] | 227 | [210;249] |
| All cancers | 204583 | [198285;211081] | 177433 | [172869;182115] |  | 89621 | [89114;90130] | 67817 | [67385;68256] |

^1^ The incidence indicators for prostate cancer relate to 2015 (last year of observation) and not 2018 (see specific comments in Materials and Methods)

**Table S5**. Estimated annual world age-standardized incidence rates with their confidence intervals by sex, cancer site, and subtype.

|  | Men | | |  | Women | | |
| --- | --- | --- | --- | --- | --- | --- | --- |
|  | Age standardized rates [95% CI] | | |  | Age standardized rates [95% CI] | | |
| Cancer site or subtype | 1990 | 2010 | 2018 |  | 1990 | 2010 | 2018 |
| *All cancers* | 320.7 [311.3; 330.5] | 370.5 [359.7; 381.6] | 330.2 [320.0; 340.8] |  | 200.6 [195.7; 205.7] | 258.7 [252.6; 265.0] | 274.0 [266.8; 281.4] |
| *Lip, oral cavity and pharynx* | 38.6 [35.0; 42.5] | 21.4 [19.4; 23.5] | 18.3 [16.6; 20.3] |  | 3.5 [3.3; 3.8] | 5.1 [4.8; 5.4] | 5.8 [5.3; 6.3] |
| *Esophagus* | 14.7 [12.9; 16.8] | 7.9 [6.9; 9.0] | 6.8 [5.9; 7.8] |  | 1.2 [1.0; 1.3] | 1.4 [1.3; 1.6] | 1.5 [1.3; 1.7] |
| Adenocarcinomas | 1.2 [1.1; 1.4] | 2.3 [2.1; 2.5] | 2.8 [2.5; 3.1] |  | 0.1 [0.1; 0.2] | 0.2 [0.2; 0.3] | 0.3 [0.2; 0.3] |
| Squamous cell carcinomas | 12.8 [11.0; 14.9] | 5.1 [4.4; 6.0] | 3.9 [3.3; 4.6] |  | 0.9 [0.8; 1.1] | 1.1 [1.0; 1.2] | 1.2 [1.0; 1.4] |
| *Stomach* | 12.2 [11.4; 13.0] | 7.6 [7.2; 8.1] | 6.3 [5.9; 6.8] |  | 4.6 [4.2; 5.0] | 3.0 [2.7; 3.2] | 2.7 [2.4; 2.9] |
| *Colon-rectum* | 40.0 [38.7; 41.4] | 38.0 [36.9; 39.2] | 34.0 [32.7; 35.3] |  | 24.0 [23.3; 24.7] | 24.0 [23.3; 24.6] | 23.9 [23.1; 24.8] |
| Colon | 22.6 [21.7; 23.6] | 22.6 [21.8; 23.5] | 20.7 [19.7; 21.7] |  | 15.1 [14.5; 15.7] | 14.9 [14.4; 15.4] | 14.8 [14.1; 15.4] |
| Rectum | 17.0 [16.3; 17.7] | 14.8 [14.2; 15.3] | 12.7 [12.1; 13.3] |  | 8.1 [7.7; 8.4] | 7.5 [7.2; 7.7] | 6.9 [6.6; 7.3] |
| Anus | 0.5 [0.5; 0.6] | 0.6 [0.6; 0.7] | 0.8 [0.7; 1.0] |  | 0.9 [0.8; 1.1] | 1.5 [1.4; 1.7] | 2.4 [2.1; 2.8] |
| *Liver* | 8.0 [7.2; 9.0] | 12.1 [10.9; 13.5] | 12.5 [11.2; 14.0] |  | 1.1 [1.0; 1.2] | 2.3 [2.1; 2.5] | 2.9 [2.6; 3.2] |
| *Pancreas* | 5.2 [4.9; 5.6] | 9.0 [8.6; 9.4] | 11.0 [10.4; 11.7] |  | 2.7 [2.5; 2.9] | 6.0 [5.6; 6.3] | 7.7 [7.2; 8.3] |
| *Larynx* | 11.6 [10.7; 12.5] | 6.0 [5.6; 6.4] | 4.8 [4.4; 5.3] |  | 0.7 [0.6; 0.7] | 0.7 [0.6; 0.7] | 0.7 [0.6; 0.7] |
| *Lung* | 51.8 [48.5; 55.2] | 51.5 [48.4; 54.9] | 50.5 [47.2; 54.0] |  | 5.4 [5.0; 5.9] | 15.7 [14.7; 16.8] | 23.2 [21.5; 25.0] |
| Adenocarcinomas | 8.9 [8.4; 9.5] | 21.6 [20.6; 22.7] | 26.2 [24.7; 27.8] |  | 1.9 [1.7; 2.1] | 8.9 [8.4; 9.5] | 15.1 [13.9; 16.3] |
| Squamous cell carcinomas | 25.9 [24.0; 28.1] | 13.8 [12.7; 14.9] | 11.3 [10.4; 12.3] |  | 1.4 [1.2; 1.6] | 1.9 [1.7; 2.1] | 2.4 [2.1; 2.8] |
| Small cell carcinomas | 7.1 [6.4; 7.7] | 5.9 [5.4; 6.4] | 5.5 [5.0; 6.1] |  | 0.8 [0.7; 0.9] | 1.9 [1.7; 2.1] | 2.7 [2.4; 3.1] |
| *Skin melanoma* | 4.8 [4.3; 5.3] | 10.9 [9.9; 12.0] | 14.2 [12.7; 15.7] |  | 6.7 [6.2; 7.3] | 11.7 [10.9; 12.6] | 14.2 [13.0; 15.4] |
| *Breast* | - | - | - |  | 72.8 [70.7; 75.0] | 95.2 [92.6; 97.8] | 99.9 [96.5; 103.4] |
| *Cervix uteris* | - | - | - |  | 10.2 [9.4; 11.0] | 6.4 [6.0; 6.9] | 6.1 [5.5; 6.7] |
| *Corpus uteri* | - | - | - |  | 10.6 [9.9; 11.3] | 11.0 [10.4; 11.7] | 11.0 [10.3; 11.8] |
| *Ovary* | - | - | - |  | 9.9 [9.4; 10.4] | 8.2 [7.8; 8.6] | 7.5 [7.0; 7.9] |
| *Prostate* | 47.2 [45.4; 49.1] | 97.3 [93.8; 101.0] | NA |  | - | - | - |
| *Testis* | 4.3 [3.9; 4.7] | 7.1 [6.6; 7.6] | 8.7 [7.9; 9.6] |  | - | - | - |
| *Kidney* | 10.6 [10.0; 11.2] | 14.8 [14.1; 15.6] | 17.1 [16.1; 18.2] |  | 4.8 [4.4; 5.1] | 6.3 [5.9; 6.7] | 7.1 [6.6; 7.6] |
| *Urinary bladder* | 17.2 [16.1; 18.3] | 14.6 [13.7; 15.5] | 14.3 [13.3; 15.3] |  | 2.5 [2.3; 2.7] | 2.3 [2.2; 2.4] | 2.4 [2.2; 2.6] |
| *Central nervous system* | 5.3 [5.0; 5.6] | 6.4 [6.1; 6.6] | 6.7 [6.3; 7.1] |  | 3.7 [3.5; 4.0] | 4.3 [4.1; 4.5] | 4.5 [4.2; 4.8] |
| *Thyroid gland* | 1.7 [1.5; 2.0] | 4.7 [4.1; 5.4] | 5.6 [4.8; 6.6] |  | 5.6 [4.8; 6.6] | 15.3 [13.1; 17.8] | 18.5 [15.8; 21.8] |

Incidence rates are expressed per 100,000 population and age-standardized to the 1960 standard world population. Incidence indicators for prostate cancer relate to 2015 (last year of observation) and not 2018 (see specific comments in Materials and Methods). Abbreviations: 95% CI, 95% confidence interval - [NC] 95% CI not calculable because the selected model does not include an effect for the year

**Table S6**. Estimated annual world age-standardized mortality rates with their confidence intervals by sex and by cancer site.

|  | Men | | |  |  | Women | | | |  | |
| --- | --- | --- | --- | --- | --- | --- | --- | --- | --- | --- | --- |
|  | Age standardized rates [95% CI] | | |  |  | Age standardized rates [95% CI] | | | |  | |
| Cancer site | 1990 | 2010 | 2018 |  |  | 1990 | 2010 | 2018 |  | |  |
| *All cancers* | 205.9 [205.2; 206.6] | 145.8 [145.4; 146.1] | 123.8 [123.0; 124.5] |  |  | 90.1 [89.7; 90.4] | 76.6 [76.4; 76.8] | 72.2 [71.7; 72.8] |  | |  |
| *Lip, oral cavity and pharynx* | 13.4 [13.2; 13.6] | 6.1 [6.1; 6.2] | 4.9 [4.7; 5.0] |  |  | 1.3 [1.3; 1.3] | 1.2 [1.1; 1.2] | 1.2 [1.1; 1.2] |  | |  |
| *Esophagus* | 11.3 [11.1; 11.4] | 5.4 [5.3; 5.4] | 4.3 [4.1; 4.4] |  |  | 1.1 [1.0; 1.1] | 1.0 [0.9; 1.0] | 1.0 [0.9; 1.0] |  | |  |
| *Stomach* | 9.0 [8.9; 9.1] | 4.7 [4.7; 4.8] | 3.9 [3.8; 4.1] |  |  | 3.6 [3.5; 3.6] | 1.8 [1.7; 1.8] | 1.5 [1.4; 1.6] |  | |  |
| *Colon-rectum* | 18.2 [18.1; 18.4] | 13.4 [13.3; 13.5] | 11.5 [11.3; 11.7] |  |  | 10.6 [10.5; 10.8] | 7.8 [7.7; 7.9] | 6.9 [6.7; 7.0] |  | |  |
| *Liver* | 10.4 [10.3; 10.6] | 9.6 [9.5; 9.7] | 9.0 [8.8; 9.2] |  |  | 2.1 [2.0; 2.1] | 2.3 [2.2; 2.3] | 2.3 [2.3; 2.4] |  | |  |
| *Pancreas* | 7.6 [7.5; 7.7] | 7.9 [7.8; 8.0] | 8.2 [8.0; 8.3] |  |  | 3.9 [3.9; 4.0] | 4.9 [4.9; 5.0] | 5.5 [5.4; 5.7] |  | |  |
| *Larynx* | 7.7 [7.6; 7.8] | 1.9 [1.9; 2.0] | 1.2 [1.2; 1.3] |  |  | 0.3 [0.3; 0.4] | 0.2 [0.2; 0.2] | 0.2 [0.2; 0.2] |  | |  |
| *Lung* | 48.2 [47.8; 48.5] | 39.5 [39.4; 39.7] | 34.7 [34.3; 35.1] |  |  | 5.3 [5.2; 5.4] | 11.1 [11.0; 11.2] | 14.0 [13.7; 14.3] |  | |  |
| *Skin melanoma* | 1.3 [1.3; 1.3] | 1.7 [1.7; 1.7] | 1.7 [1.6; 1.8] |  |  | 1.0 [1.0; 1.0] | 1.1 [1.0; 1.1] | 1.0 [1.0; 1.1] |  | |  |
| *Breast* |  |  |  |  |  | 20.2 [20.0; 20.3] | 16.0 [15.9; 16.1] | 14.0 [13.8; 14.2] |  | |  |
| *Cervix uteri* |  |  |  |  |  | 3.1 [3.1; 3.2] | 1.9 [1.9; 1.9] | 1.7 [1.6; 1.8] |  | |  |
| *Corpus uteri* |  |  |  |  |  | 2.7 [2.6; 2.7] | 2.3 [2.2; 2.3] | 2.3 [2.3; 2.4] |  | |  |
| *Ovary* |  |  |  |  |  | 6.0 [5.9; 6.1] | 4.4 [4.4; 4.5] | 3.9 [3.8; 4.0] |  | |  |
| *Prostate* | 18.1 [17.9; 18.2] | 10.8 [10.7; 10.8] | 7.9 [7.8; 8.1] |  |  |  |  |  |  | |  |
| *Testis* | 0.4 [0.4; 0.4] | 0.2 [0.2; 0.3] | 0.2 [0.2; 0.3] |  |  |  |  |  |  | |  |
| *Kidney* | 4.6 [4.6; 4.7] | 4.5 [4.5; 4.6] | 5.0 [4.9; 5.2] |  |  | 1.8 [1.8; 1.9] | 1.5 [1.5; 1.6] | 1.5 [1.5; 1.6] |  | |  |
| *Urinary bladder* | 7.0 [6.9; 7.1] | 5.3 [5.2; 5.3] | 4.7 [4.6; 4.8] |  |  | 1.3 [1.3; 1.3] | 1.0 [1.0; 1.0] | 0.9 [0.9; 1.0] |  | |  |
| *Central nervous system* | 3.9 [3.8; 4.0] | 4.0 [3.9; 4.0] | 4.3 [4.1; 4.4] |  |  | 2.5 [2.4; 2.6] | 2.5 [2.5; 2.6] | 2.7 [2.6; 2.8] |  | |  |
| *Thyroid gland* | 0.4 [0.4; 0.4] | 0.3 [0.2; 0.3] | 0.2 [0.2; 0.2] |  |  | 0.5 [0.5; 0.5] | 0.2 [0.2; 0.3] | 0.2 [0.2; 0.2] |  | |  |

Mortality rates are expressed per 100,000 population and age-standardized to the 1960 standard world population. Deaths rates from cervix uteri and corpus uteri cancers were re-estimated (see specific comments in Materials and Methods). Abbreviations: 95% CI, 95% confidence interval - [NC] 95% CI not calculable because the selected model does not include an effect for the year


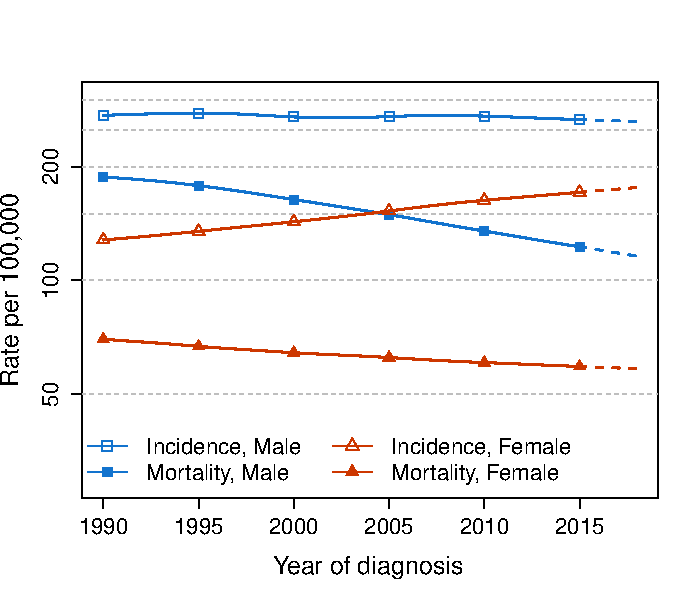


**Supplementary Figure S1**. Trends in age-standardized cancer incidence and mortality rates (log-scale) by sex, 1990-2018, France (all cancers excluding prostate and breast cancers).
